# Meeting--Mad River

**Published:** 1883-06

**Authors:** 


### Meeting--Mad River.

The annual meeting of the Mad River Dental Society was held according to announcement in Dayton on the 22nd of May. There were a goodly number in attendance. The occasion was one of much interest. The beginning was prompt at the time appointed, and the time was fully used till the moment of closing. The subjects considered were practical and full of interest, and discussed in such a manner as to bring out the prominent features of each one, particularly was this true of setting of artificial crowns upon roots of teeth; the good methods were clearly described, and the doubtful or defective ones were lightly passed over.

The question pertaining to empiricism was freely discussed, and doubtless with profit to all present. This statement will certainly be emphasized to every reader of the brief article of Prof. Wright on page 293 of this number. This phase of the subject is an exceedingly important one, and should receive the attention not only of dentists, but of the people in general, and our legislators in particular. It is very desirable that the profession should move in this direction. The only regret we have in respect to this meeting is for the poor fellows who ought to have been there, and were not.

